# Detection of Potato ring rot Pathogen *Clavibacter michiganensis* subsp. s*epedonicus* by Loop-mediated isothermal amplification (LAMP) assay

**DOI:** 10.1038/s41598-019-56680-9

**Published:** 2019-12-31

**Authors:** Hasan Sagcan, Neslihan Turgut Kara

**Affiliations:** 1 Istanbul University, Institute of Science, Program of Molecular Biology and Genetics, Istanbul, Turkey; 2 Istanbul University, Faculty of Science, Department of Molecular Biology and Genetics, 34134 Istanbul, Turkey

**Keywords:** DNA probes, Assay systems

## Abstract

*Clavibacter michiganensis* subsp. *sepedonicus* (CMS) is an important bacterial plant pathogen causing potato ring rot disease. Rapid diagnosis of CMS is crucial because of the economic losses caused by serious harvest losses. Although there are serological tests used in the rapid diagnosis of CMS, they are not widely used because of their low sensitivity. The DNA-based PCR methods, which are highly sensitive, do not have the possibility of on-site diagnosis, especially since they require serious laboratory infrastructure. In recent years, scientists have been working on alternative amplification methods to develop DNA-based point of care (POC) diagnostic methods. Accordingly, the loop-mediated isothermal amplification (LAMP) method, which was developed in the early 2000s, provides an important convenience for DNA-based tests to use in the field. Due to the unique design of primers, more amplification products could be create in a shorter time than conventional amplification methods without needing a temperature cycle, and it can be applied with the aid of a simple heater without requiring a laboratory environment. In this study, efficient LAMP method for the detection of CMS has optimized. For device-independent detection of LAMP products, colorimetric method and LFD has used.

## Introduction

Potato ring rot is a quarantine pathogen that leads to major economic losses caused by *Clavibacter michiganensis* subsp. *sepedonicus* (CMS). The only organism in which CMS naturally causes infection is potato. Symptoms of the disease generally appear at the end of the growing period. The first symptoms are seen in the lower leaves and leaves first turn a light green color, then gray and brown, and necrotic structures are formed. On the tubers, there are brown cracks with red edges^1^. CMS is a pathogen that cannot be controlled easily because it can remain viable on the surface of equipment and materials used in potato production. One of the most important causes of the spread of potato ring rot disease is the use of potato seeds contaminated with CMS, making CMS-free seed production very important in the eradication of the pathogen^2^. Therefore, reliable and sensitive methods to detect potato ring rot pathogen are needed. Several serological methods, such as immunofluorescence assay (IFA) and enzyme-linked immunosorbent assays (ELISA), including were developed for the detection of CMS^3^. However, there are some limitations in the use of serological methods that they are low sensitivity (>10000 CFU ml^−1^) and open to cross-contamination^4^.

In recent years, DNA-based molecular methods have been frequently used in the diagnosis of plant pathogens^5–8^. Polymerase chain reaction (PCR) is undoubtedly the most commonly used method of DNA-based diagnostic tests^9,10^. PCR methods are used for the detection of CMS in many studies^11–13^. Mills^14^ reported that the limit of detection was 100 CFU ml^−1^ using PCR methods. This result shows that PCR methods are approximately 100 times more sensitive than serological methods. Even if conventional PCR and real-time PCR have certain advantages such as high sensitivity compared to other methods, they have serious disadvantages^15,16^. Requiring expensive and complex devices and experts, and long analysis times are important limitations^17^.

There is a need for a DNA based molecular diagnostic method for the diagnosis of CMS that is suitable for point of care (POC) tests making it viable for use in the field and customs area. At this point, DNA amplification methods that are required for laboratory infrastructure are not suitable for POC tests^18,19^. However, the loop-mediated isothermal amplification (LAMP) method developed by Notomi^20^ provides innovation for DNA-based tests to be used as field tests^21–24^. Due to its unique primer design and Bst DNA polymerase, the reaction does not require a temperature cycle, and can be carried out on a simple heater at a constant temperature. In the LAMP reaction, pairs of inner (FIP, BIP) and outer (F3, B3) primers are used. Each of the inner primers contains a complementary sequence region (F2, B2) and the same sequence region (F1c, B1c) on the target sequence. After the F2/B2 region of the inner primer is attached to the target gene, the elongation reaction begins. The loop structure forms by the attachment of the F1c/B1c region of the inner primer to the complementary region (F1, B1) in the new product, which is released with the help of outer primers. Free inner primers are bound to this loop region and the reaction becomes continuous^25,26^. Because it uses 4 or 6 different primers that recognize 6 or 8 different regions on the target gene, the LAMP method is more sensitive than other PCR methods^27–29^. The LAMP method is preferred to use in the field diagnosis of CMS because it is relatively simple, fast, highly specific and also does not require complex laboratory devices during application^30^.

One of the innovations offered by the LAMP method is that the amplification products can be detected without the need for complex imaging devices^30^. Positive reactions can be detected by measuring the turbidity of the magnesium pyrophosphate, which is excessively exposed during the reaction, using pH indicator dyes and the lateral flow test strips^31–33^. The lateral flow test strips used to visualize LAMP products contain biotin ligand as the test line and anti-rabbit antibody to the control line. The FIP primer designed for detection is labeled with biotin, and a probe with the FAM label is designed. Probe hybridization was performed after the LAMP reaction and results visualized with the help of lateral flow test buffer. In the positive samples, one side of the reaction products will be biotin-labeled and the other part will be FAM-labeled. Positive lamp products attach to the test line with the help of biotin ligand, and gold nanoparticles that bind to the anti-FAM antibody will attach to the other part of the positive LAMP product^34^, thus, providing detection via colorimetric reaction. All these detection methods facilitate the use of the LAMP method with point-of-care tests and microfluidic chips^35^.

The aim of this study was to optimize the LAMP method for *Clavibacter michiganensis* subsp. *sepedonicus* to obtain a simple and fast diagnosis. Besides, positive samples were demonstrated by colorimetric and lateral flow test strip methods for detection without the need for any imaging devices. After using simple DNA isolation methods and lyophilization of reaction mixtures, a suitable method for field studies could be obtained.

## Materials and Methods

### Samples and DNA isolation methods

To prevent the spread of pathogens within the country, only CMS (NCPPB No. 4229) DNA in closed tubes was obtained from Istanbul Directorate of Agricultural Quarantine, where it was purified by the bacteriology laboratory. The DNA of other pathogens [*Escherichia coli* (25922)*, Salmonella spp* (ATCC 14028)*, Listeria monocytogenes* (ATCC 7644)*, Clostridium perfringens* (ATCC 13124)*, Staphylococcus aureus* (ATCC 25923)] was purified using QIAGEN QIAamp DNA mini kit, according to the manufacturer’s instructions. CMS-free *Solanum tuberosum* DNA, which was used as the negative control, was purified using SureFood PREP Advanced Kit (CONGEN, S1053) according to the manufacturer’s instructions. Isolated DNA samples were first analyzed by Nanodrop 2000 (Thermo Fisher Scientific, Waltham, MA) and gel electrophoresis. DNA quality was determined by the A_260_/A_280_ ratio.

### LAMP primer and probe design

Specific LAMP primers of CMS and FAM labeled-probe, which was used in detection of LAMP products in the diagnostic method with LFD, were designed using PrimerExplorer V5 according to the 16 S rDNA intergenic spacer region (GenBank accession no: AF001266.1). Details of primers and the probe are shown in Table 1.

**Table 1:** LAMP primers and probes (Turkish Patent Office, Patent application No. 2018/19039) targeting the 16 S rDNA intergenic spacer region of *Clavibacter michiganensis* subsp. *sepedonicus* (GenBank accession no: AF001266.1).

Primer	Sequence (5′-3′)
F3	GCGCGATAGAAGAGGAACTC
B3	GGACATCTCTCAGGTGCCA
FIP (F1c-F2)	GCGGACATTCAAGGACCGAGG-CGTGATCAAGGAAGTCGTCG
BIP (B1c-B2)	CAGGTCACCACGGTACTGAGC-GTCCTGAGCAACGACAAGA
Probe	FAM-GGCTTTTGCCAGATT

### LAMP assay

LAMP reactions were performed on Biorad (T100) and Amplyus (miniPCR) devices using the *Bst* 3.0 DNA polymerase enzyme kit (NEB, M0374L) with a total volume of 25 µl. The reaction used 0.2 µM for each outer primers (F3 and B3), 0.8 µM for each inner primers (FIP and BIP), 1X reaction buffer [20 mM Tris-HCl, 10 mM (NH_4_)_2_SO_4_, 2 mM MgSO_4_, 0.1% Tween^®^ 20, pH 8.8 at 25 °C], 4 mM MgSO_4_, 0.4 mM, 1 mM and 1.5 mM dNTP (three different concentrations were tested for optimization), 2 U Bst polymerase and 5 µl DNA (up to 30 ng µl^−1^ concentration) sample. Reactions were incubated at 65.6 °C, 67 °C, 68 °C, 68.8 °C, 69.2 °C, 70 °C and 71.4 °C °C for 60 min, followed by incubation at 80 °C for 10 min for enzyme denaturation. LAMP products were analyzed by 2% w/v agarose gel electrophoresis and observed by UV-transilluminator.

### Visualization of LAMP products

The visualization of LAMP products was conducted by three methods. In the first one agarose gel (2% w/v) electrophoresis was used to evaluate LAMP products. 1X TAE buffer was used to run LAMP products on the gel. The samples were run at 90 V for 60 minutes and the gel was observed on UV-transilluminator (Avegene-Xlite 30 R). In the second one, a FAM labeled-probe was used for lateral flow dipstick (LFD-Milenia Biotec-Hybridetect) detection performed according to the manufacturer’s instructions. After the LAMP reactions, 20 pmole FAM labeled-probe was added and incubated for 5 min 65 °C. At the end of hybridization, 8 µl hybridized product was transferred to 100 µl assay buffer. Finally, a test strip was dipped into the final product and the result was observed in 5 min. In the final method, colorimetric assay, NEB warmstart colorimetric LAMP 2X master mix kit was used according to the manufacturer’s instructions. The colorimetric assay was performed using 2X colorimetric LAMP buffer, 0.2 µM F3-B3 primers, 0.8 µM FIP-BIP primers, and 5 µl DNA (10 ng µl^−1^) with a total volume of 25 µl. The reaction was incubated at 65 °C for 30 minutes as indicated in the kit protocol. Results were evaluated according to color formation. In the positive reaction, the color in the wells turns yellow, in the negative reaction there is no color change and the tubes appear pink.

### Sensitivity and specificity of LAMP assay

The LAMP assay’s sensitivity in finding the lowest detectable amount of CMS DNA was examined using 10-fold serial dilutions of pure isolated CMS DNA^36^. For this, 10 ng µl^−1^, 1 ng µl^−1^, 100 pg µl^−1^, 10 pg µl^−1^, 1 pg µl^−1^ and 0.1 pg µl^−1^ CMS DNA were tested. The specificity of the LAMP assay based on the 16 S rDNA IGS region was determined using *Solanum tuberosum* and 5 different pathogens: *Escherichia coli, Salmonella spp., Listeria monocytogenes, Clostridium perfringens*, and *Staphylococcus aureus*. The reactions were carried out under the conditions resulting from the optimization of the LAMP method. LAMP sensitivity and specificity were analyzed by 2% w/v agarose gel electrophoresis.

### Ethical approval

This article does not contain any studies with human participants or animals performed by any of the authors.

## Results

### LAMP primer and probes

Specific LAMP primers of CMS were designed using PrimerExplorer V5 software. Primers were specific to the 224 bp region of 16 S rDNA IGS. Also, the probe was selected outside of the primary regions in target sequences. It was determined that primers given by the V5 program did not have any specificity against any subtypes of *Clavibacter michiganensis*.

### LAMP assay

Three different concentrations of dNTP were tested for the optimization of the LAMP assay and were incubated at seven different temperatures for optimization of the reactions. The optimum temperature for the reaction was found to be 70 °C and the most suitable dNTP concentration was found to be 1.5 mM.

### Visualization of LAMP product

In this study, LAMP products were visualized using the gel electrophoresis method, lateral flow dipstick, and colorimetric LAMP kit.

**Figure 1:**
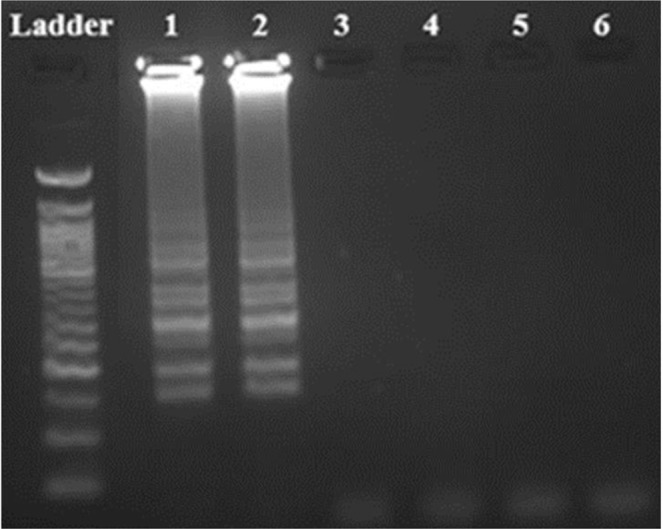
Visualization by agarose gel electrophoresis of optimized LAMP assays. Ladder: 50 bp (NEB, N0473), Lane 1: CMS 10 ng µl^−1^, Lane 2: CMS 10 ng µl^−1^, Lane 3: *Solanum tuberosum* 10 ng µl^−1^, Lane 4: *Solanum tuberosum* 10 ng µl^−1^, Lane 5: No template, Lane 6: No template.

In agarose gel electrophoresis, ladder-like band formation which refers to the positive reaction in the LAMP method, was observed for CMS samples. Ladder-like band formation was not observed in the negative control well (Fig. 1).

**Figure 2:**
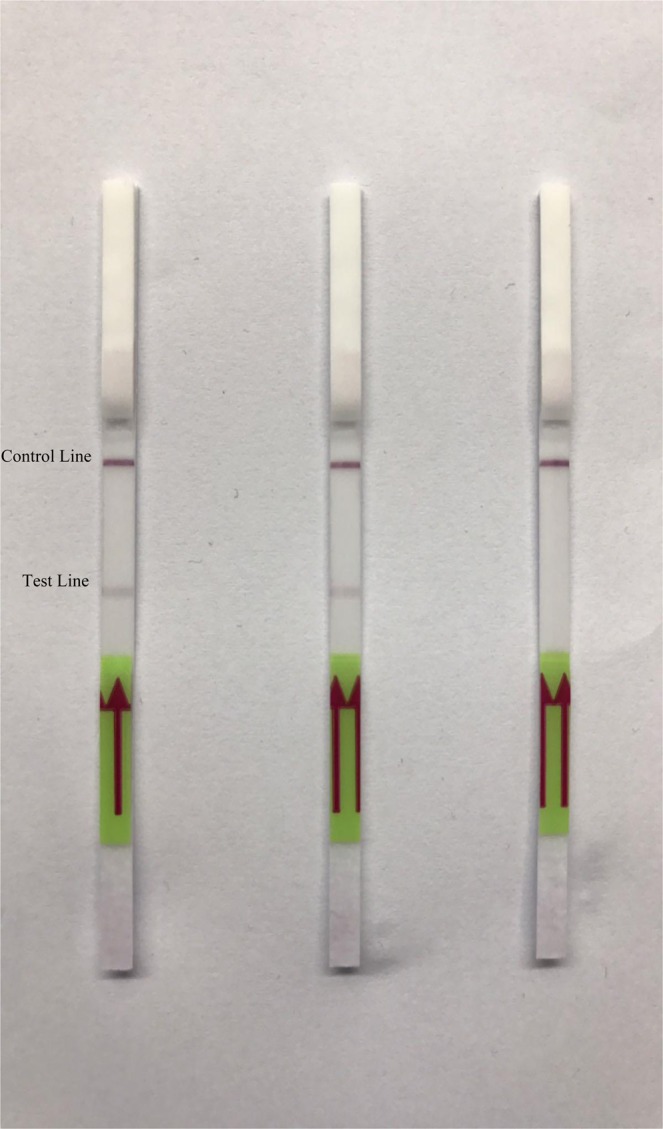
Visualization by lateral flow dipstick. LFD 1: CMS 30 ng µl^−1^, LFD 2: CMS 10 ng µl^−1^, LFD 3: No template (from left to right).

In visualization with LFD, test lines were detected in CMS samples but not in negative samples. The test lines in the 30 ng µl ^−1^ sample and in the 10 ng µl^−1^ sample have almost the same significance. Finally, the formation of control lines in all samples showed that the lateral flow test strips functioned correctly (Fig. 2).

**Figure 3:**
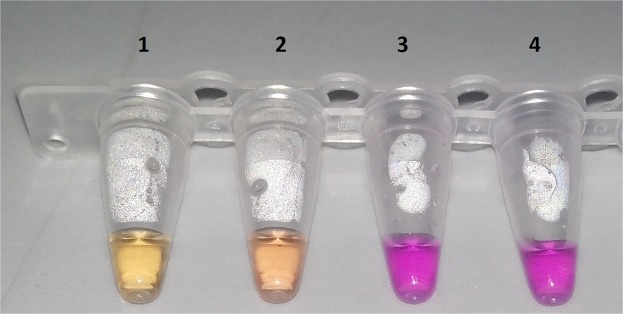
Visualization by colorimetric method. Tube 1: CMS 30 ng µl^−1^, tube 2: CMS 10 ng µl^−1^, tube 3: No template, tube 4: No template.

In the colorimetric assay, while CMS samples turned yellow, no color changes were detected in the negative controls. There were color differences between the 30 ng μl^−1^ and 10 ng μl^−1^ samples, proving that more LAMP products were formed in the 30 ng μl^−1^ sample. As a result, the increase in the amount of DNA in the positive sample showed indicated a color closer to yellow in the reaction tube (Fig. 3).

### Sensitivity and specificity of LAMP Assay

**Figure 4:**
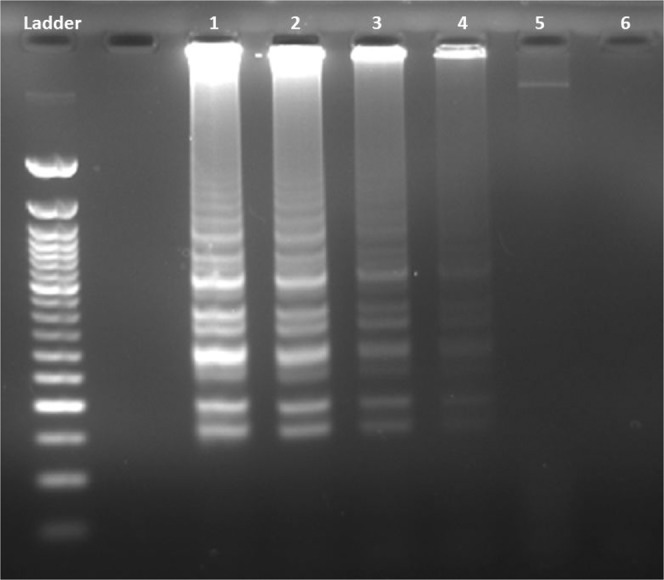
The sensitivity of the optimized LAMP assay. Ladder: 50 bp (NEB, N0473), Lane 1: 10 ng µl^−1^, Lane 2: 1 ng µl^−1^, Lane 3: 100 pg µl^−1^, Lane 4: 10 pg µl^−1^, Lane 5: 1 pg µl^−1^, Lane 6: 0.1 pg µl^−1^.

Serial dilutions of the CMS samples were prepared to measure the sensitivity of LAMP primers. CMS DNA was studied in six different dilutions between 10 ng µl^−1^ and 0.1 pg µl^−1^. As a result, it was determined that the minimum amount of CMS DNA needed for diagnosis, or in other words the sensitivity of the method, was 10 pg µl^−1^ (Fig. 4).

**Figure 5:**
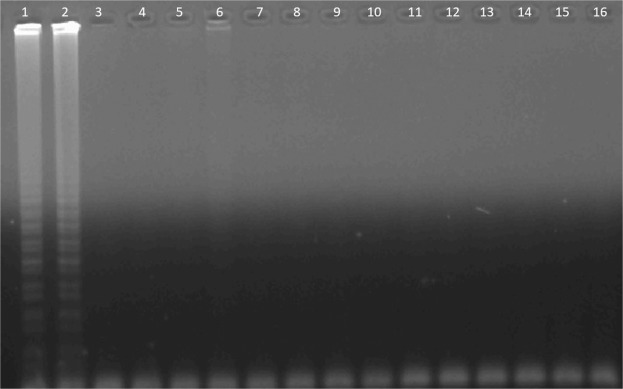
The specificity of the optimized LAMP assay. Lane 1–2: CMS 10 ng µl^−1^, Lane 3–4: *Solanum tuberosum* 10 ng µl^−1^, Lane 5–6: *Escherichia coli* 10 ng µl^−1^, Lane 7–8: *Salmonella* spp. 10 ng µl^−1^, Lane 9–10: *Listeria monocytogenes* 10 ng µl^−1^, Lane 11–12: *Clostridium perfringens* 10 ng µl^−1^, Lane 13–14: *Staphylococcus aureus* 10 ng µl^−1^, Lane 15–16: No template.

For the specificity of lamp primers, we checked for any matchup with different pathogens. In this direction, *Escherichia coli, Salmonella spp., Listeria monocytogenes, Clostridium perfringens*, and *Staphylococcus aureus, Solanum tuberosum* samples were used. The primers did not give positive results, except CMS (Fig. 5).

## Discussion

Potato ring rot disease, which results in serious damage, is caused by *Clavibacter michiganensis* subsp. *sepedonicus* which is listed as a quarantine pathogen in the European and Mediterranean Plant Protection Organization (EPPO) A2 list and in EU plant health legislation^37^. This bacterial pathogen causes serious economic losses in potato production worldwide. For this reason, rapid and sensitive diagnosis of transmission is important to prevent losses. However, *Clavibacter michiganensis* subsp. s*epedonicus* diagnostic methods, still commonly used especially in national laboratories, are time consuming. In this study, a rapid, sensitive and easily applicable method for the diagnosis of *Clavibacter michiganensis* subsp. s*epedonicus* was developed. LAMP method optimization for 9 different subspecies of *Clavibacter michiganensis* was performed in the study of Dobhal *et al*.^38^. However, because the method recognizes all types of *Clavibacter michiganensis*, it will show positive results for other sub-species non-pathogenic on potato. For this reason, additional analysis is necessary when detecting CMS using this method. Another restriction of the study by Dobhal *et al*.^38^ is that LAMP products are not suitable for field use because they are detected by laboratory devices. In our study, the LAMP method for CMS was developed and no further analysis was needed to detect this potato pathogen. Our results are also suitable for field studies because the amplification products could be determined by colorimetric or lateral flow test strips.

Optimization of the LAMP method has become widely studied in the last decade. In this study, we performed LAMP for the detection of *Clavibacter michiganensis* subsp. *sepedonicus*, which has damaging effects on the potato plant. Many methods such as gel electrophoresis, SYBR green, turbidity detection, calcein staining, lateral flow test strips (LFD), colorimetric LAMP method can be used for the detection of LAMP products^39–43^. For the detection of *Clavibacter michiganensis* subsp. *sepedonicus* LAMP products, we optimized the gel electrophoresis method, lateral flow test strips and colorimetric LAMP methods. Lateral flow strips and the colorimetric LAMP method are especially suitable for diagnosis of *C. michiganensis* subsp. *sepedonicus* in the field because neither of these visualization methods need any device besides simple and portable amplification devices such as MiniPCR (Amplyus, USA) and Genie II (Optigene, UK).

LAMP is a method that rapidly multiplies the target nucleic acid sequence at isothermal conditions^44^. The LAMP method is more specific than other types of PCR due to the use of 4 to 6 different primers that specifically recognize 6 to 8 distinct regions of the target gene^27–29^. Generally, 50 times more amplicon can be produced in the LAMP method than other PCR types^45^. Although there are many different isothermal amplification methods besides LAMP, most of them are not suitable for the amplification of large DNA sequences^46^. Moreover, the LAMP method can be easily integrated into microchip diagnostic devices (Lab-on-a-chip), sensor-based devices and POC diagnostic devices^47^.

Amplification temperature is the most important parameter of nucleic acid-based diagnostic methods. Incorrectly determined amplification temperature may cause both false positivity and false negativity^48^. Therefore, amplification temperature experiments should be studied repetitively and carefully. Amplification temperature was detected to be 63 °C in the LAMP method reported by Li *et al*.^49^ developed for GMO analysis. In the LAMP method optimization study for detection of maize chlorotic mottle virus (MCMV), 6 different amplification temperatures in the range of 60 to 65 °C were tried and the optimum amplification temperature was determined to be 63 °C^50^. Again, in many publications, the amplification temperature was found to be 65 °C and below. However, in our studies, false positivity was found at temperatures of 65 °C and below. For this reason, temperatures up to 71.4 °C were used for optimization of the amplification reaction. As a result, the optimal amplification temperature for the diagnosis of *Clavibacter michiganensis* subsp. *sepedonicus* was determined to be 70 °C.

In general, the sensitivity of the LAMP method is higher than other PCR-based methods^27–29^. In our study, the lowest detectable amount of CMS DNA by the developed method was found to be 10 pg µl^−1^, which is equal to 1.36 × 10^4^ copy of CMS DNA. In other words, we performed 10-fold serial dilutions with three biological replicates and two technical replicates, and the lowest amount of template that gave positive amplification was 1.36 × 10^4^ copy DNA. This value is the lowest detectable amount of CMS DNA that can be detected in all replications of the sensitivity studies. In addition, in the study by Cho *et al*.^13^, the sensitivity of CMS detection with a real-time PCR method was found to be 5 fg. But according to the CMS detection guide issued by EPPO, the reference method can detect 10^3^–10^4^ cell ml^−1^ CMS, which means at least 10^4^ copy DNA, and it is also the typical bacterial load for CMS on potato plants^51^. Based on this information, it is assumed that the sensitivity value of our method is sufficient. Zhang *et al*.^36^ reported that the lowest detection limit was characterized using 10-fold serial dilutions of pure *F*. *fujikuroi* DNA (1 ng to 10 ag) extract. The sensitivity was confirmed on plant samples. Even if the sensitivity of the developed LAMP method is lower than the real-time PCR method, the LAMP method has many advantages such as being rapid, simple and cost-effective.

Consequently, the rapid and sensitive diagnosis of plant diseases is very important to prevent serious harvest losses. As is clearly seen in the EPPO guide^51^, rapid diagnostic test methods which constitute the first step of the CMS detection and identification workflow are really important to prevent the spread of these kind of pathogens. But these rapid diagnostic methods, such as IF, FISH or PCR, depend on complex and expensive laboratory infrastructure. This study optimized the LAMP method, which is a more simple, affordable, user-friendly, sensitive and faster method than current plant disease diagnosis methods, for CMS. We also determined the feasibility of detecting plant diseases independent from laboratory infrastructure: using LAMP products with the lateral flow test strip and the colorimetric LAMP method, in addition to gel electrophoresis.
